# Musical trends and predictability of success in contemporary songs in and out of the top charts

**DOI:** 10.1098/rsos.171274

**Published:** 2018-05-16

**Authors:** Myra Interiano, Kamyar Kazemi, Lijia Wang, Jienian Yang, Zhaoxia Yu, Natalia L. Komarova

**Affiliations:** 1 Department of Mathematics, University of California Irvine, Irvine, CA 92697, USA; 2 Department of Statistics, University of California Irvine, Irvine, CA 92697, USA; 3 Department of Ecology and Evolutionary Biology, University of California Irvine, Irvine, CA 92697, USA

**Keywords:** music evolution, complex social dynamics, temporal trends

## Abstract

We analyse more than 500 000 songs released in the UK between 1985 and 2015 to understand the dynamics of success (defined as ‘making it’ into the top charts), correlate success with acoustic features and explore the predictability of success. Several multi-decadal trends have been uncovered. For example, there is a clear downward trend in ‘happiness’ and ‘brightness’, as well as a slight upward trend in ‘sadness’. Furthermore, songs are becoming less ‘male’. Interestingly, successful songs exhibit their own distinct dynamics. In particular, they tend to be ‘happier’, more ‘party-like’, less ‘relaxed’ and more ‘female’ than most. The difference between successful and average songs is not straightforward. In the context of some features, successful songs pre-empt the dynamics of all songs, and in others they tend to reflect the past. We used random forests to predict the success of songs, first based on their acoustic features, and then adding the ‘superstar’ variable (informing us whether the song’s artist had appeared in the top charts in the near past). This allowed quantification of the contribution of purely musical characteristics in the songs’ success, and suggested the time scale of fashion dynamics in popular music.

## 1. Introduction

Music, and, in particular, songs, rarely leave people unmoved. There is something magical about music and scientists have been trying to disentangle the magic and explain what it is that makes us love some music, hate other music and just listen to music [1–3]. In particular, it has been noticed that different generations like different types of music. In this exploratory study, we focus on the popular songs of the last three decades, and attempt to extract multi-decadal trends in the musical attributes of both successful and less successful songs, and to see what it is that makes songs popular in different years.

A number of authors have investigated the popularity of songs and analysed the dynamics of top charts. Different correlates of success have been identified and studied. One of the earlier studies [4] focused on the relationship between the song’s position in the charts and the duration of time that the song stayed in the charts, and found that greatest hits (that is, songs that scored highest) remained in the charts for longer periods. Bhattacharjee *et al*. [5] developed a stochastic model of the distribution of album longevity in the Billboard Top 100 Chart. It was found that, since the advent of file-sharing networks and related market changes, the probability of songs’ survival in the charts decreased, and the albums that do survive then have much improved prospects. Buda & Jarynowski [6] investigated trends in songs’ popularity in 12 western European countries by analysing data from weekly charts between 1966 and 2015. They found that geographical and cultural distances played a different role in songs’ success in different eras (analogue, digital and Internet).

Many different factors have been studied in connection with songs’ top chart dynamics, including popularity (‘superstar’) status and the demographics of the artist(s), the level of promotion, the label/company, etc; these factors could be roughly classified as ‘socioeconomic’ factors (as opposed to ‘musical’ factors). Asai [7] studied hits in Japanese popular music during two 3-month periods (between January and March) in 1990 and in 2004, before and after the penetration of the Internet and multi-channel broadcasting. The authors conducted a parametric survival analysis (the exponential model) and examined different variables involved in producing a hit: ‘company’ (whether a song is produced by one of five major companies), ‘genre’ (whether or not the song is J-pop), ‘star’ (whether the artist’s sales last year reached a certain value), ‘tie’ (whether a single is tied with other media for promotion purposes) and ‘multi-artist’ (whether an album is a compilation featuring several albums). It was found that fame of the artist, ties with other media and compilation albums featuring several artists prolonged the hit’s chart period. A survival analysis was also used in [8], which measured the statistical correlations of the period that an album stayed in the weekly hit charts in the US music market. They found that the rank at which an album debuts in the hit charts, the artist’s career, promotion via major or minor labels, and the artist’s gender influenced the hit charts’ longevity.

Lee [9] also studied top chart dynamics, but focused on a different type of question. Lee investigated changes in the diversity of songs by examining the number of songs of different genres entering top 10 charts each year, and found that song diversity declined significantly in all genres between 1992 and 2002. In a more recent study Ordanini & Nunes [10] investigated song dynamics in the US Billboard Hot 100 singles chart between 1974 and 2013, and consistent with [9] found a decline in the number of songs in both the top 10 and top 100 from 1974 until about 2003 (the winner-takes-all effect). Afterwards, however, the trend reversed itself as the number of songs making the chart increased steadily after the launch of legitimate online music sellers such as iTunes. The exact opposite pattern was observed for artists. The authors concluded that this overall pattern reflected a transition from fewer blockbusters by more superstars to more blockbusters by fewer superstars.

Apart from socioeconomic factors, the influence of the musical characteristics of songs on their chart dynamics has also been studied. Mauch *et al*. [11] considered the evolution of popular music by using the US Billboard Hot 100 charts between 1960 and 2010, and analysed the musical properties of 17 000 recordings by using data-mining tools and measuring a series of quantitative audio features. Analysis of musical diversity revealed that, according to a number of different measures, musical diversity has not declined, and also that the gradual evolution of musical styles has been punctuated by a number of ‘revolutions’. Askin & Mauskapf [12] also studied musical features of songs. They processed Billboard’s Hot 100 in 1958–2013. Two types of characteristics of songs were investigated: (i) data from discogs.com, a crowd-sourced but community-monitored site containing extensive artist, album and track data, and (ii) the Echo Nest, an online music intelligence provider that assigns to each song its audio characteristics, such as tempo, loudness and key, as well as features like ‘danceability’, ‘acousticness’, etc. Their statistical analysis found that several of the musical characteristics of songs correlate with the songs’ success (measured as reaching the top 1 or top 10 in the charts, or remaining in the charts for a long time). The authors then went on to explore cultural networks of songs (defined by the patterns formed by the songs’ musical attributes) and found that songs that are too similar to their neighbours have a harder time moving up the charts, unless a song is especially unique, in which case it could benefit from not being crowded by other similar songs.

In this paper we focus on songs’ success, and study how this depends on the musical characteristics of the hits. In contrast with most of the papers mentioned above, we study a much larger class of songs, both in and out of the charts. This allows us to define songs’ success simply as ‘making it’ into the charts. This is a cruder definition of success than the definitions used previously, such as the songs’ longevity or peak position in the top charts. The advantage of this approach is that including songs both in and out of the top charts allows for more variation in musical characteristics and thus endows them with a larger predictive power. Our goal is to understand the dynamics of success, correlate success with musical features, and explore the predictability of success in the future by using past trends. We analyse more than 500 000 songs released in the UK between 1985 and 2015, and use statistical and machine learning approaches in order to determine (i) to what degree actual musical attributes of songs contribute to the songs’ success and (ii) what musical trends can be observed in contemporary music over the last 30 years.

## 2. Data collection

### 2.1. Sources

Among the myriad ways of gauging a song’s popularity are sales, airplay, video streaming, Twitter mentions and Internet searches. Owing to a long, consistent and available history of record keeping, trade magazine charts are a logical first step in attempting to build a model of musical preferences over time. For our study, we chose the Top 100 Singles Chart by the Official Charts Company in the UK. We then extended these data by pairing as many songs as possible with metadata and musical feature descriptors from MusicBrainz and AcousticBrainz (see below).

#### 2.1.1. UK charts

The pre-eminent industry source for pop music rankings in the UK is the Official Singles Chart released by the Official Charts Company [13]. The company makes available an online database that stretches back to the 1950s, but the charts it contains actually span several companies which sometimes used different information sources or different methodologies. Further, the charts were different lengths at different times, but, with one small exception, have featured 100 songs since 1983. The database represents what the current owners, who took over in 1994, feel is the most consistent historical lineage for the charts. The present-day stewards of the chart are the British Phonographic Industry, representing the British recording business; the Entertainment Retailers Association, representing digital and physical music retailers and audio and video streaming services; and the Millward Market Research Company, which compiles the sales figures. The top 100 rankings are based on sales of downloads, compact discs, vinyl and audio streams. Digital downloads were incorporated into the rankings in 2004, audio streams in 2014 and, in 2007, the requirement that a song has a physically available equivalent was dropped.

#### 2.1.2. MusicBrainz

MusicBrainz is a community-generated public repository of music metadata accessible over the Internet or as a static download [14]. It began its life in the 1990s as an open source alternative for compact disc identification. It has since widened its scope by expanding the information it maintains for releases and tracks to include associated information such as genre, country of origin, date of release and artist aliases. The expanded nature of the information, available in the form of a searchable database, allows for the possibility of uncovering more complicated relationships that can be used in music research and music discovery. The primary means of organization for the database are a series of unique identifiers, referred to as MBIDs (MusicBrainz identifiers), which tag certain aspects of the data.

#### 2.1.3. AcousticBrainz

AcousticBrainz is a project that aims to democratize and facilitate music research by making more comprehensive data freely available to the public [15]. It is the brainchild of Xavier Serra of the Music Technology Group (MTG) at the Universitat Pompeu Fabra in Barcelona, Spain, in collaboration with MusicBrainz. Historically, copyright and logistical issues have made it difficult for researchers to access large collections of musical information with which to test hypotheses and create algorithms. At times large databases have been made available such as the Million Song Dataset and the commercial repository the Echo Nest, but the Million Song Dataset is not updated to reflect new music and the Echo Nest has limitations on its use and no access to the underlying algorithms. AcousticBrainz’s answer to these challenges has been to develop a ready-made framework that can be used to crowdsource data from individual music libraries.

There are three main components of the AcousticBrainz framework. The first one is its feature extraction. At the core of the AcousticBrainz efforts to build a repository of information within the limits of copyright is a library of feature extraction algorithms called Essentia. Although the libraries are available in a user-modifiable form for the generation of new algorithms using the gathered information, what AcousticBrainz has done is package them together in the form of pre-compiled binaries to facilitate ease of use for volunteers and ensure that the data are obtained and calculated in a uniform manner. Individuals contribute to the project by downloading the suite of algorithms, running them on their music collections and returning the data to the project. The software scans the collection and outputs low-level acoustic information such as spectral, time domain, rhythm and tonal descriptors which include information like beats per minute and song duration. It is this step that comprises the second component of AcousticBrainz as that of a data storage hub. The low-level information collected is available to anyone for testing and development of algorithms without needing access to a huge collection of music. Finally, the third component of AcousticBrainz is the creation of semantic musical descriptors. The returned data are analysed using machine learning methods and classification techniques trained on annotated datasets, and these high-level features are added to the information contained about each song.

### 2.2. Features

The high-level musical features in the AcousticBrainz database are obtained by applying classifiers, trained on sets of songs annotated by experts, to the low-level information users’ upload; the characteristics of the variables are described online [15]. Users have the ability to train their own models, but, for the sake of consistency, only high-level features calculated using a pretrained set of classifiers are disseminated to the public. Based on researchers’ experiences with the current data and users’ creation of new models, the classifiers will probably be updated in the future as the accuracy of the models’ ability to make predictions on larger datasets is unknown.

There are a total of 18 variables corresponding to the high-level features calculated by AcousticBrainz used in this analysis, 12 of which are binary and six of which are categorical. Each variable has an estimated posterior probability of accuracy associated with it. For example, for binary variables, each song was either classified as having or not having the binary feature, and along with this a posterior probability of the classification of being correct was provided. The binary variables are broadly broken up into two categories: the acoustic properties of the music (such as timbre or danceability) and the moods describing the sounds (such as sad, party or happy). The 12 binary variables are presented below. The first five are acoustic properties of music, followed by two sound characteristics, and five moods.

1. timbre—colour—(dark/bright)
2. tonality—(tonal/atonal)
3. danceability—(danceable/not danceable)
4. voice, instrumental—(voice/instrumental)
5. gender—gender in vocal music—(male/female)
6. mood, acoustic—sound type—(acoustic/not acoustic)
7. mood, electronic—sound type—(electronic/non-electronic)
8. mood, relaxed—(relaxed/not relaxed)
9. mood, sad—(sad/not sad)
10. mood, party—(party/not party)
11. mood, happy—(happy/not happy)
12. mood, aggressive—(aggressive/not aggressive)

There also six categorical variables which attempt to categorize the music into more complex mood and genre classes.

1. moods, mirex:
  — Cluster1: passionate, rousing, confident, boisterous, rowdy
  — Cluster2: rollicking, cheerful, fun, sweet, amiable/good-natured
  — Cluster3: literate, poignant, wistful, bittersweet, autumnal, brooding
  — Cluster4: humorous, silly, campy, quirky, whimsical, witty, wry
  — Cluster5: aggressive, fiery, tense/anxious, intense, volatile, visceral
2. genre, electronic—ambient, dnb, house, techno, trance
3. genre, tzanetakis—blues, classical, country, dis, hip-hop, jazz, met, pop, reg, rock
4. genre, dortmund—alternative, blues, electronic, folk, country, funk, soul, rhythm and blues, jazz, pop, rap, hip-hop, rock
5. genre, rosamerica—classical, dance, hip-hop, jazz, pop, rhythm and blues, rock, spe
6. rhythm, ismir04—cha cha, jive, quickstep, rumba, samba, tango, Viennese waltz, slow waltz

For each of these six categorical classifications, each song is assigned one category (together with a probability of it being an accurate description). In particular, for ‘1. moods, mirex’, one of the five clusters is assigned to each song. Similarly, for ‘2. genre, electronic’, one of the five categories is chosen.

The four different genre variables above differ from each other by the methodology of classification; see [16]. They are based on models and algorithms developed by different groups. The first one (‘genre, electronic’) is a dataset developed by the MTG for the purpose of labelling songs as one of five different electronic music categories. The second one (‘genre, tzanetakis’) is based on the work of George Tzanetakis and is described in [17]. Based on this work, an open source software framework for audio processing and music information retrieval (MIR) is provided online [18]. The third (‘genre, dortmund’) is based on work done at TU Dortmund University described in [19] and [20]. The data are available online [21]. Finally, ‘genre, rosamerica’ was also developed by the MTG and is described in [22,23].

The two remaining categorical variables owe their names to abbreviated community titles. The International Society of Music Information Retrieval (ISMIR) is a non-profit group that organizes a yearly conference that includes data challenges, where all participants receive the same labelled training dataset [24]. In 2004, one of the challenges was to develop an algorithm for automatic tempo classification. The rhythm classifier (‘6. rhythm, ismir04’) provided by MusicBrainz is based on these data. Finally, the Music Information Retrieval Evaluation eXchange (MIREX) is a community-based framework for evaluating MIR systems and algorithms [25]. Like ISMIR, the MIREX community issues yearly challenges and datasets for different MIR tasks. In 2009 the group issued the task of mood categorization. The categories to be adopted were those described in categorical variable ‘1. moods, mirex’. Songs used in the training set for this classifier were manually assigned by humans [26].

The 12 binary features listed above are used in §3 to study multi-decadal trends and signatures of success; also, categorical variable ‘5. genres, rosamerica’ is used to control for the genres. All the variables, both binary and categorical, are used in §4 to predict songs’ success.

### 2.3. Data extraction

The Official Charts Top 100 data begin on the week of 6 January 1985 and end on the week of 19 July 2015, representing 148 190 entries and 30 081 unique songs. As the Official Charts Company does not provide the public with an application programming interface to download data, we used Python scripts to scrape the information from the published charts and create a searchable database.

A static copy of the MusicBrainz database was downloaded on 19 April 2016 from its ftp site.^1^ The database, along with its schema, is made available on its website and illustrates all the relationships that can be searched for. The information we used from MusicBrainz is organized by alphanumeric identifiers (referred to as MBIDs) that correspond to each release. According to the MusicBrainz documentation,^2^ ‘a release is the unique issuing of a product available for consumption, as distinguished by characteristics such as date, country and packaging’, the first two being the most salient for our purposes. Therefore, the same song released in the USA would have a different MBID from the equivalent song released in the UK.

The AcousticBrainz public data have been released as a series of 19 incremental updates each split into high-level and low-level data. The low-level data are the set of acoustic information returned to the project by users. The high-level information is the set of semantic descriptors attached to each song by trained classifiers. A copy of the original AcousticBrainz data dump from January 2015 and the 18 incremental data dumps were downloaded on 22 April 2016, representing 1 641 765 unique songs. The same release MBIDs that organize the MusicBrainz data also organize the AcousticBrainz data. Each update is a series of JSON files, each of which corresponds to a release MBID of the song it belongs to and holds all the information from the Essentia musical descriptor extraction and metadata from MusicBrainz associated with it.

There is no mechanism, as of now, for checking for duplicate submissions. It may be that different users submit features for the same song, with the same MBID to AcousticBrainz. This is potentially a problem, because the musical descriptors generated by the software can be affected by the quality of the source recording or other factors. For consistency, we kept only the first occurrence of any song.

Because we wanted to compare successful songs with unsuccessful songs, we needed to identify the candidate pool of unsuccessful songs. Generally, because of the different rules that were in effect throughout the history of the chart, it is difficult to create a set of non-charting songs in a uniform way which would always reflect the rules of the chart at the moment they were released. For this study, for the non-charting list we decided to include all available songs from a UK-distributed release (albums, singles, EPs, vinyl, etc.). In fact, for the present-day rules, this is not so arbitrary as any song available for paid download is theoretically eligible for the charts. Once candidate songs were identified, we retrieved their musical descriptors from the AcousticBrainz database we created from the publicly released data.

Extracting the music features for charting songs was not as straightforward. To begin with, there are many more non-charting songs than charting songs. As AcousticBrainz is an open source database dependent on the submissions of volunteers, and country-specific releases of identical songs may not be represented, for charting songs we did not require that the music descriptor information come from a release tagged as UK specific, as long as it matched the title and artist. Another problem is that the data are from two different sources, the Official Charts Company and MusicBrainz. Hence there are variations in spelling, word order and artist name convention that made reconciling records difficult. One of the most problematic areas was songs with multiple collaborators. To try to mitigate the effects of differences in artist listings, we tokenized artist names and ignored joining phrases. We were able to match 13 073 unique songs exactly without any data manipulation. Furthermore, we were able to match an additional 1463 songs after tokenizing the artists’ names and using the natural language processing capabilities of MySql, for a total of 14 536 songs out of 30 081 total unique charting songs.

As the database in AcousticBrainz is populated by volunteers, it is possible that a bias exists because of preferences of the people who submit songs. It must be noted, however, that, by construction, the bias (if any) is the same for both charting and non-charting songs. To check for the existence of a bias, one would need to compare the feature distribution of the songs in the database with the feature distribution of all songs (or a large superset of songs). Unfortunately, such information was not available to us. AcousticBrainz was the largest database with relevant information that we had access to. Nonetheless, in order to obtain a rough idea of the extent of a possible bias, we performed the following pilot calculation. Focusing on the data for 2005, we compiled a list of all the charting songs (1189 songs) and compared it with the list of charting 2005 songs that were included in the analysis (678 songs that we were able to match with AcousticBrainz). To compare the two sets, we randomly picked 100 songs from each of the two lists; we refer to the resulting sets of 100 songs as set A and set B, respectively. For both of these sets, we identified the genre of all the songs by looking up each song manually. If more than one genre was listed, we used the first one in the list. Note that the genre assignments collected in this way are different from the categorical genre variables in AcousticBrainz (§s2b), which we used everywhere else in this paper.

We compared the three largest genre categories, which were rock, alternative/indie and pop. Pearson’s *χ*^2^ test with Yates’ continuity correction revealed that the differences are not statistically significant. This analysis suggests that if a bias exists between the set used in this work and the set of all songs, it is not a large bias.

**Figure 1.**
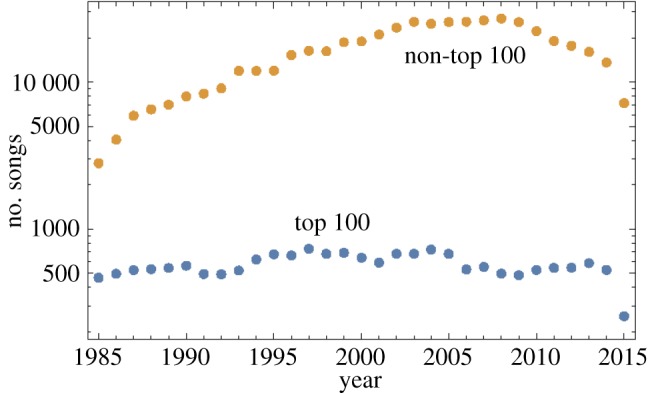
The number of top 100 and non-top 100 songs with feature information available, for 1985–2015.

Figure 1 shows the number of songs (with feature information available) that have been extracted, between 1985 and 2015. The information for 2015 at the time of extraction was incomplete, and therefore this year has fewer songs. For all other years, on average 582 top 100 songs per year were extracted, with the range from 466 in 1985 to 733 in 1997. On average, 16 245 non-top 100 songs were extracted per year with the range from 2805 in 1985 to 27 095 in 2008.

## 3. Data analysis

### 3.1. The concept of success

The success of contemporary songs can be defined in a variety of ways. For example, Rossman [27] studied the radio airplay time as a correlate of songs’ success; and Dertouzos [28] focused on the economic aspects of success. In an insightful paper [12], the top chart performance was studied, and two measures of songs’ success were explored: (i) the maximum song popularity was defined as its peak chart position, and (ii) as a measure of sustained popularity, the number of weeks in the charts was used. While correlations between various musical features and success have been identified by Askin & Mauskapf [12], their analysis has been necessarily limited by the fact that only the charted songs were considered, while most songs were left out of the calculations. In the current work, we consider both charting and non-charting songs (the latter is the great majority of songs). Further, we define the success of a song simply as ‘making it’ into the charts.

### 3.2. Temporal trends in songs’ features

**Figure 2.**
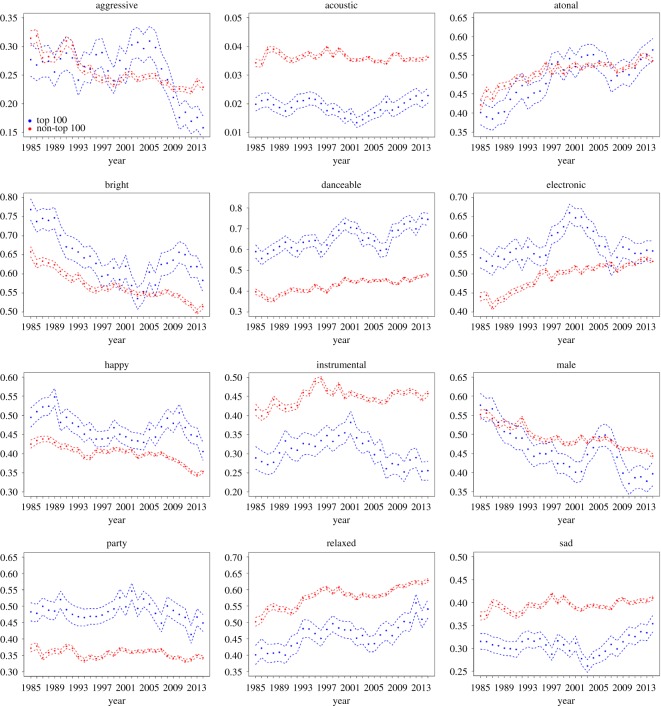
The mean values for the 12 features of top 100 songs (blue dots) and non-top 100 songs (red dots) along with their 95% confidence intervals (dashed lines) using *t*-distribution, for 1985–2014.

Figure 2 shows the temporal trends in average songs’ 12 features along with their confidence intervals, both for non-top 100 songs collected (red dots) and top 100 songs (blue dots). To create this figure, each of the 12 binary variables was converted into a value in [0,1] by using the posterior probabilities. For example, if the variable ‘happy’ is 1 for a given song, and its posterior probability is *p*, then the value is given by *p*; if the variable is 0, then the value is 1−*p*. Then the means and the 95% confidence intervals were calculated for each variable and for each year. From figure 2, we can see that, for the majority of features, the mean features of successful songs are very different from those of uncharted songs, suggesting that successful songs follow their own distinct pattern. The significance remains even after adjusting for multiple tests.

Apart from suggesting that successful songs constitute a separate class of songs (see more details below), figure 2 also informs us about the temporal trends that occurred in the songs’ features over the last 30 years. First we note that, overall, ‘happiness’ and ‘brightness’ experienced a downward trend, while ‘sadness’ increased in the last 30 or so years. This is consistent with the findings of [29], who examined the lyrical content of popular songs in 1980–2007. It was found that the use of positive emotion in songs had dwindled over time. In particular, it was reported that popular music lyrics now include more words related to a focus on the self (e.g. singular first person pronouns), fewer words describing companionship and social contact (e.g. plural first person nouns) and more anti-social words (e.g. ‘hate’, ‘kill’, etc.). The authors explain these trends as being in tune with the overall increase in tendencies towards loneliness, social isolation and psychopathology [30,31].

Second, ‘relaxedness’ and ‘danceability’ increased in the last three decades (with a possibly related increase in ‘electronic’ and ‘atonal’ characteristics). A related interesting study of cultural tendencies in music is presented by Straw [32], who examined the phenomenon of polarization of contemporary (1990s) popular music between dance-based pop and rock/heavy metal. The author describes a certain decline in the mainstream rock music, quoting several reasons, including the changes in the political role of music, the absence of new performers and lack of innovation in rock music since punk. He then proceeds to note that the act of dancing is ‘intimately bound up with a generalized sense of diminished inhibition’. This hints at a possible correlation between the upward trends in danceability and relaxedness that is observed in figure 2.

Finally, we can see that the percentage of male voices experienced a downward trend. Moreover, we observe that successful songs are characterized by a larger percentage of female artists compared with all songs. We comment on these trends in the next section.

### 3.3. Signatures of success

As mentioned in the previous section, the mean characteristics of successful songs often differ systematically from those of all songs; see figure 2. In particular, we observe the following patterns:

(1) Successful songs are happier than average songs.
(2) Successful songs have a brighter timbre than average songs.
(3) Successful songs are less sad than average songs.
(4) Successful songs are more party-like than average songs.
(5) Successful songs are less relaxed than average songs.
(6) Successful songs are more danceable than average songs.

Patterns (1–5) are very interesting because, with respect to these five features, popular songs do not reflect the overall tendency. While one might expect the majority of popular songs to be in the vanguard, this does not appear to be the case. As was described above, the majority of songs experienced a decline in happiness and brightness and an increase in sadness (with the underlying tendency in increased negativity). The successful songs seem to defy these trends. One reason that could contribute to this tendency is the growing success of compilation albums [33], which have proved to be very popular as a risk-reducing strategy based on the repackaging and reintroducing of older songs.^3^ Another component could be the growing importance of older music consumers [33] that may introduce certain ‘inertia’ in the success of popular music.

Also, there are some new distinctions that seem to be emerging in the last decade or so:

(7) In recent years, successful songs are more often sung by females. This is particularly interesting given a large debate about the role of women in the music industry [34], especially the issues of gender inequality, stereotypes and the sexualization of female singers [35].
(8) In recent years, successful songs have been less aggressive than average songs, perhaps suggesting a partial reversal of the trends described in [29].

**Figure 3.**
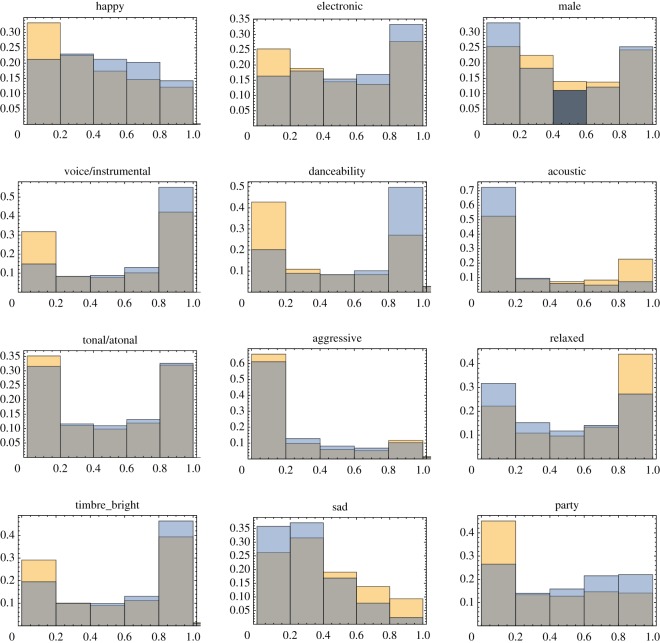
Distributions of different features of successful (blue) and unsuccessful (yellow) songs. The intersection of the two histograms appears grey in the figure. The cumulative data over the 30 years are shown. Individual histograms for each year show similar patterns (not shown).

These patterns are also observed in figure 3, where we plotted probability distributions for all the features for successful (blue) and unsuccessful (yellow) songs. Again, the values obtained by using the posterior probabilities have been used. The differences in the distributions are highly significant for many features.

### 3.4. Genre and success

**Figure 4.**
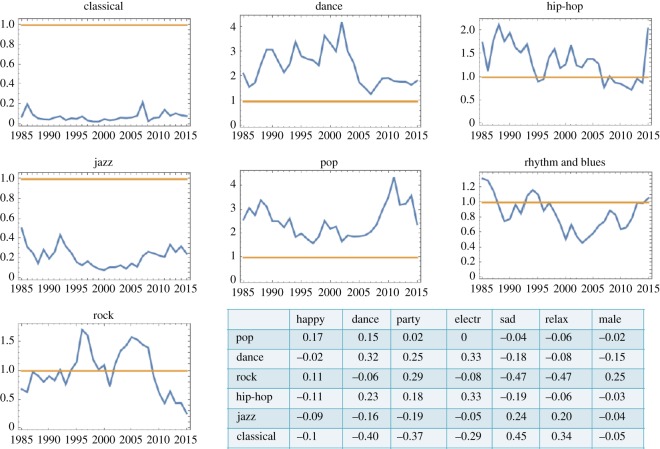
The statistics of the genres. The odds ratios for different song genres are presented for 1985–2015. In the table, Pearson correlation coefficients between genres and features are shown.

For completeness we investigated the influence of different genres on songs’ success. The genres according to the ‘rosamerica’ methodology were used [16], because this method gives the most balanced genre representation in the dataset. In figure 4 we present the odds ratios of seven of the genres. One can see that classical and jazz songs are unlikely to be successful (in the narrow definition used in this study), while dance and pop are the most successful of the genres. We also observe a clear downward trend in the success of rock starting in the early 2000s. In the table at the bottom of figure 4 we present the Pearson correlation coefficients between the six most represented genres and some of the musical characteristics, for which clear trends were observed in figure 2. Clearly, genres and features are not independent, and one can see many correlations that are hardly surprising, such as a positive correlation between the genre ‘dance’ and the feature ‘party’, and a negative correlation between the genre ‘rock’ and the feature ‘relaxed’.

To understand the connection between genre and success, we repeated the analysis of figure 2, while controlling for the songs’ genre. The results are intuitive. If, within a genre, a given feature has a reduced variation compared with its variation among all songs, this feature does not differ strongly between successful and unsuccessful songs within that genre. For example, if we only consider songs with genre ‘dance’, their danceability feature is very high, in both successful and unsuccessful songs, and we do not observe a significant difference between successful and unsuccessful songs in the context of this feature. Many trends however persist when controlling for the genre. For example, among ‘pop’ songs, successful songs are characterized by higher ‘danceability’, and they are more ‘party-like’ and ‘electronic’; further, among ‘rock’ songs, successful songs tend to be more ‘happy’, more ‘danceable’, more ‘party-like’ and less ‘relaxed’, the same trends as shown in figure 2. Interestingly, the successful ‘rock’ songs are more ‘male’ than non-successful ones, which is the opposite of the overall trend. This can possibly be understood from the specifics of the genre, where more male singers have historically been successful. There are three more such reversal of trends that we observed: ‘rock’, ‘jazz’ and ‘classical’ songs are less ‘electronic’ when successful (whereas, in general, successful songs tend to be more ‘electronic’). This is possibly because this feature is strongly present in the more popular genres, such as ‘pop’ and ‘dance’, but it does not help the popularity of these other, non-electronic genres.

We note finally that although the analysis of success in the context of the genre is interesting, it has to be understood that automatic determination of genre is a difficult task, and there are a lot of inconsistencies between the four genre variables in AcousticBrainz. Therefore, it appears that the lower-level variables, such as the 12 binary musical characteristics listed in §s2b, provide more information about the trends in songs’ success.

## 4. Prediction of success

Given that multiple features show significant differences between successful and non-successful songs, we next examine whether a song’s success can be predicted using songs published in earlier years. Specifically, we use random forests to make predictions for the songs in three different years: 2014, the most recent year with a full-year data; 2004, the year with two-thirds of the data that can be used; and 1994, the year with one-third of the data that can be used. Random forests are a predictive method based on classification trees, which are known as a flexible predictive tool to model linear and nonlinear functions of features. Compared with the classification tree method, which relies on a single tree, the method of random forests overcomes overfitting by combining predictions from a list of decision trees, which are created by random resampling of observations and subsampling covariates/features [36]. The R package ‘RandomForest’ is used in our analysis. We also considered other classification methods, such as single-layer and two-layer neural networks. For our data, we found that they gave similar performance to random forests. As a result, they will not be reported here.

### 4.1. Predicting songs’ success in 2014 using random forests

#### 4.1.1. Scheme 1: Random forest model trained on each year

For each year between 1985 and 2013 (inclusive), we trained a random forest model using data from each year to predict if a song from 2014 will be top-charted. As the population of the music data is highly unbalanced (i.e. there were many more non-charted songs than top-ranking songs), we only sampled a subset of non-charted songs (whose size is equal to the number of charted songs) along with all charted songs of a year to train a model. For example, we sampled 585 out of 16 058 non-charted songs of 2013 because there were 585 top-charted songs for that year in the dataset. We applied the same sampling method to create the test set using the data from 2014. For 2014, there were 526 top-charted and 13 592 non-charted songs. We used all top-charted songs and sampled 526 out of 13 592 non-charted songs for the test set. When we tested the prediction performance of each trained model, we fixed the test set. That is, we only sampled the test set once. We employed the accuracy rate to measure the prediction power, which is defined as the percentage of the times the correct class (top-charted or non-charted in this case) is predicted for songs in the test set. We experimented with this scheme in the context of types of approaches. For the first approach, we used 18 categorical variables with levels described in §s2b as explanatory variables. For the second approach, we converted the first 12 binary features out of the 18 categorical variables into a positive number between 0 and 1 using the probability associated with the level of a feature, and kept other multi-level categorical variables as they were described in §s2b. We then trained and tested the model using the 12 continuous variables (with values ranging between 0 and 1) and the six multi-level categorical variables. For the following schemes, we only focused on the first approach.

#### 4.1.2. Scheme 2: Combined random forest model

Motivated by the power of ensemble learning, we combined individual random forest models trained in the previous scheme into one model using weights proportional to individual prediction accuracy obtained in scheme 1 with feature probabilities. That is, employing all individual models, the prediction of the class probability for instance *j* (i.e. the probability that song *j* from 2014 will be top-charted), $y_{j}^{c}$, using the combined model is

$$\begin{array}{rl} y_{j}^{c} & =\sum_{i=1985}^{2013}w^{\left(i\right)}\cdot y_{j}^{\left(i\right)},\\ w^{\left(i\right)} & =\frac{{\mathrm{acc}}_{i}}{\sum_{i=1985}^{2013}{\mathrm{acc}}_{i}}, \end{array}$$

where *acc*_*i*_ and $y_{j}^{\left(i\right)}$ are the prediction accuracy and the class probability for instance *j* using the random forest model trained on year *i* (obtained by scheme 1), respectively. We assigned a test case *j* to the class top-charted if $y_{j}^{c}>0.5$ and to the class non-charted otherwise. Note that we used feature probabilities rather than binary features because we found that the former method produces higher prediction accuracies.

#### 4.1.3. Scheme 3: Random forest model trained on all (or a sub-collection of) data

Different from scheme 2, which combines models, this scheme combines data. Specifically, we trained a random forest model with time as a continuous variable, using all the data (or a subset) of songs from 1985 to 2013 (inclusive). We then tested the prediction performance of the model on 2014. The training and testing sets were collected according to the procedure described in scheme 1.

### 4.2. Including the ‘superstar’ variable

**Figure 5.**
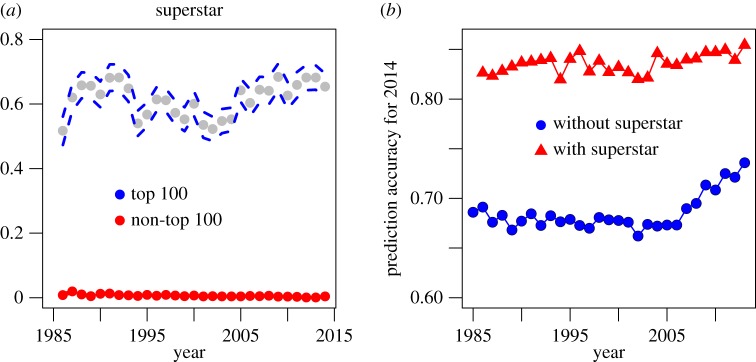
Proportions of superstar artists and prediction accuracies for songs in 2014. (*a*) Proportions of superstar artists among charted and non-charted songs from 1986 to 2014. Because data before 1985 were not available, superstars were not defined for artists in 1985. (*b*) Prediction accuracies using scheme 1 with continuous input. The figure shows the average accuracy from 10 simulations per year. The *x*-axis is the year of the training data.

The concept of ‘superstars’ has been identified as an important factor in determining the success of songs. It was suggested by Adler [37] that only a relatively small number of artists and their products reach enormous success, because consumers minimize the cost of search by simply choosing artists who are already popular. We call an artist a superstar if at least one song of the artist was top-charted in the previous year (definition 1) or in the past 5 years (definition 2). A preliminary analysis showed that the superstar variable defined by definition 2 has a much higher predictability; as a result, we used the 5 year definition in the subsequent analysis. Among the charted songs, the proportions of songs with a superstar artist were between 0.51 and 0.68; see figure 5*b*. Note that the lowest proportion occurred in 1986, which is due to the fact that only songs in 1985 can be used to define superstars. Among the non-charted songs, the proportions of songs with a superstar artist are much lower—between 0.0009 and 0.02. This big difference indicates that superstar might be a variable of high predictive value. To examine this hypothesis, we repeated the analysis above including the superstar variable as a predictor.

### 4.3. Predictions for 2004 and 1994

To examine whether conclusions in predicting songs from 2014 can be generalized to other years, we investigated whether songs in two other years (2004 and 1994) can be predicted successfully using songs in previous years. The predictive models we considered are similar to those used to predict songs in 2014.

### 4.4. Results for random forest models

Table 1 and figure 5*b* summarize the prediction performance of songs from 2014 (averaged over 10 simulations) of various models using the above schemes. The result shows that individual random forest models trained on 2013 produce better prediction accuracy than other individual models trained on a different year. Moreover, models trained on recent years (2010–2013) perform better than others in terms of prediction accuracy. This is particularly true when the superstar variable was not used. When the superstar variable was included in the predictive analysis, however, this trend is weakened. Note that superstar is a strong predictor for success, as suggested by the substantial difference between the two accuracy curves in figure 5*b* and between the columns in table 1. For example, when using continuous inputs of songs in 2013 to predict songs in 2014, including the superstar variable improves the accuracy of prediction by 15% (0.85 versus 0.74). We observe that the superstar variable, being such a strong predictor of success, masks subtler trends in the prediction based on musical features: while the blue line in figure 5*b* shows a decrease towards past years, the red line in figure 5*b* has a much slower decrease. Note that the superstar variable values are independent of the horizontal axis, as they are calculated based on the artists’ performance in the previous 5 years.

**Table 1.** Prediction performance for songs in 2014, with and without the superstar variable, by using different schemes.

	without superstar	with superstar
sch 1: categorical input	yr 2013: 0.71 (best)	yr 2013: 0.82 (best)
sch 1: continuous input	yr 2013: 0.74 (best)	yr 2013: 0.85 (best)
sch 2 trained w/yr 2009–2013	0.73	0.86
sch 2 trained w/yr 2004–2013	0.73	0.85
sch 2 trained with all years	0.69	0.86
sch 3 trained w/yr 2009–2013	0.75	0.85
sch 3 trained w/yr 2004–2013	0.70	0.86
sch 3 trained with all years	0.70	0.85

From the results shown in table 1, we also observe that the model based on 2013 is comparable, in terms of prediction accuracy, to methods that combine models or previous years’ data, and training with more but earlier data does not improve prediction accuracy.

**Figure 6.**
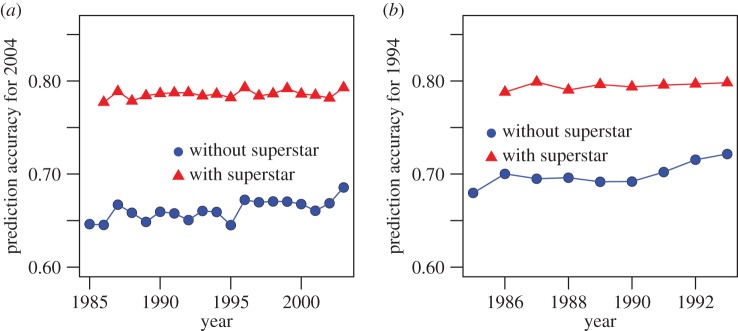
Prediction accuracies for songs in 2004 (*a*) and 1994 (*b*).

To examine whether the trends identified for 2014 are observed for other years, we presented the prediction accuracies for years 1994 and 2004 (figure 6). Consistent with results for 2014, superstar is also an important predictor for songs from 1994 and 2004. Compared with the results for 2014, the prediction accuracies for 1994 and 2004 are lower and the increasing trend is less obvious. One possible explanation is that the data quality of late years, especially those after 2005, are much better, as a result of the boom in online music sales.

## 5. Discussion and conclusion

In this study, we used the extensive data from the UK charts, MusicBrainz and AcousticBrainz, which included more than 500 000 songs released over the last 30 years (1985–2015), to study the dynamics of song success. We defined a song as a success if it appeared in the charts. We studied the influence of the musical characteristics of the songs and attempted to find correlates of songs’ success, as well as explore the multi-decadal trends in the music industry. While the features used in this study are defined by availability in the large public database, one can argue that the present study has a theoretical value. Very similar musical features were used in [12], where they were found to be correlated with songs’ success (which was measured either as longevity in the charts or the top position in the charts); these features, however, did not have sufficient power to predict success. In our analysis over a larger class of songs and with a cruder definition of success (as ‘making it into the charts), we were able to create reasonable predictive models, by using the random forests method. So, from this perspective, one can say that the result obtained suggests that the features are reasonable.

Moreover, many of the features that are used in the analysis (e.g. the ‘moods’) are shown to obey a distinctive trend over the last several decades, which agrees with trends reported by researchers in other areas of cognitive and social and behavioural studies. This brings us to the first result reported here, which concerns the uncovered temporal trends; see figure 2, red lines. A clear downward trend in ‘happiness’ and ‘brightness’ as well as a slight upward trend in ‘sadness’ are consistent with the findings of DeWall *et al*. [29], who studied multi-decadal trends in songs’ lyrics, and found that the use of positive emotions has declined over the years, and semantic indicators of loneliness and social isolation have increased in frequency. It is interesting that, in this particular instance, acoustic characteristics of songs indicate similar patterns to those uncovered in lyrics, because, in general, the relationship between songs’ melodic and lyrical contents is quite complicated [38]. Further, our study has found a multi-decadal increase in songs’ ‘danceability’ and ‘relaxedness’, which may indicate a gradual increase in the popularity and spread of dance-based pop compared with the opposing rock-type song style [32]. Finally, we find that the ‘maleness’ of the songs (that is, the frequency of male singers in popular music) has decreased over the last 30 years. Remarkably, the successful songs have shown a tendency to be even less ‘male’ than the average songs in the most recent years studied.

This brings us to the second set of results obtained in this analysis, which is the comparison between average songs and successful songs. First we note that successful songs (i.e. charted songs) are relatively rare. It has been reported that only 2% of albums marketed in the USA during 1998 sold more than 50 000 copies and only 10% of all titles are able to break even [33]. In our dataset, on average, less than 4% of all songs are successful each year. By comparing acoustic characteristics of songs, we have shown that successful songs are quite different from the majority of songs and their features sometimes follow their own trends. This can be clearly seen from figure 2, by comparing the blue lines (successful songs) with the red lines (unsuccessful songs). We observed that successful songs are happier, brighter, more party-like, more danceable and less sad than most songs. Some of these trends point to the tendency of most successful songs to go against the general multi-decadal trends. The public seem to prefer happier songs, even though more and more unhappy songs are being released each year.

Further, we noticed that, while successful songs’ trends more or less mirror the general trends (often at a different quantitative level), there are two qualitatively distinct relationships between the successful song features and the overall multi-decadal dynamics. (i) For two of the features (‘danceability’ and ‘male’), the successful songs seem to predict the overall tendency. For example, in the context of ‘danceability’, successful songs are more ‘danceable’ than average songs, and the overall trend is an increase in ‘danceability’. (ii) For four of the features, it appears that the successful songs reflect the ‘past’ of average songs. For example, while the overall ‘happiness’ of both successful songs and average songs has been decreasing over the last 30 years, successful songs are much ‘happier’ than unsuccessful ones.

This underlies the general notion that success is difficult to define and generalize. While Rentfrow & Gosling [39] found a clear relationship between people’s personality features and their musical preferences, in [40], it was shown that people have difficulties predicting their own future likes and dislikes. Further, quoting [41], ‘Hit songs, books and movies are many times more successful than average, suggesting that “the best” alternatives are qualitatively different from “the rest”; yet experts routinely fail to predict which products will succeed’. In our case, even though we can see that, in general, successful songs are, for example, ‘happier’, more ‘party-like’, less ‘relaxed’ and more ‘female’ than most, this does not necessarily allow us to naively predict that a particular ‘happy, party-like, not relaxed’ song sung by a female is going to succeed; the same conclusion as was reached by [12]. Therefore, we have attempted to use more sophisticated, machine learning methods to see if we can predict success with any degree of reliability. It turned out that

(i) using the acoustic characteristics available to us, we were not able to exceed a prediction accuracy of 0.74;
(ii) using information in the past did not improve the prediction; in other words, only the previous year’s data, and not older data, seemed to contribute significantly to our ability to predict successfully in a given year;
(iii) by adding a non-acoustic feature (the ‘superstar’ status), we were able to attain a prediction accuracy of 0.86.

These findings are quite interesting. With regard to point (i) above, our inability to exceed the 0.74 mark of the prediction accuracy (with acoustic features alone) speaks to the existence of missing information. Note that this limit was obtained when we focused exclusively on the musical features of songs, and those are of course not the whole story, as demonstrated clearly in point (iii) above. It has been shown in the past that other factors contribute significantly; in fact, some authors such as Asai [7] investigated success as a function of only non-musical factors. Other authors studied the relationship between musical and social factors. In [41] the authors simulated a musical marketplace to show that popular appeal is determined by both social influence and artistic quality. The authors concluded that ‘Success was also only partly determined by quality: The best songs rarely did poorly, and the worst rarely did well, but any other result was possible.’ Hamlen [42] investigated the merits of two opposing views on the reasons for success. One view is that the consumers of popular music have no recognition of or appreciation for ‘quality’ or ‘ability’ in singing. The second view states that the ‘superstar phenomenon’ dominates the dynamics of success, where very small differences in ability get magnified into disproportional levels of success. These authors investigated specifically the correlation between an objectively measured ‘voice quality’ and success and found that, although it did not explain it completely, it played a significant role in the success of songs.

Our results, as expressed in (i) and (iii) above, suggest a quantitative measure for these patterns. While we found that the prediction accuracy can be greatly improved when the superstar variable is included, the fact that we are able to predict success with an accuracy of 0.74 based on acoustic features only suggests that truly musical characteristics are very important. Our analysis quantifies exactly the extent of this importance in the last decades of popular music.

Point (ii) above suggests that musical fashion is relatively short-lived. It has been proposed in the literature that fashion in general is complex and fashion changes are difficult to explain. Quoting Lieberson [43], ‘fashion has its own internal mechanisms that generate changes even in the absence of societal change…. By definition, fashion changes simply for the sake of fashion—a constant stream of new features and designs is a must for many products’; see also [44]. It has been suggested that the fast dynamics of fashion is driven by the relationship between fashion and social identity and manifestation of the status hierarchy. While many people in a group tend to engage in copying behaviour, those at the top of the hierarchy attempt to differentiate themselves from those below by acting as trendsetters [45,46]. Another factor that may be responsible for the fast dynamics of change is the fierce competition among the music suppliers in the overcrowded market, and their strife to ‘stand out in the crowd’ [47].

In general, our exploratory investigation into the dynamics of popular music and songs’ success revealed that an analysis of acoustic features allows us to identify a number of multi-decadal trends, defines successful songs with their own dynamics and helps (to a certain degree) predict songs’ success.

## Footnotes

1 http://ftp.musicbrainz.org/pub/musicbrainz/data/fullexport/.

2 https://musicbrainz.org/doc/Release.

3 Although we define success as simply making it into the chart, it must be remembered that this is still in the context of a particular year. Were this not the case, then we would expect that re-issues of previous hits would always chart, but the data do not support this; only some songs manage to chart again. Thus, what is being claimed is that the difference in the characteristics of re-issued songs that manage to chart again versus old hits that do not represent something important either about the intrinsic nature of songs that manage to be hits even in new years or about the tastes of the demographics whose musical consumption habits are most represented by the hit criteria of the chart.

## Data accessibility

The data used in this paper are publicly available, as explained in the text and specified in the references, and also listed below: the Official Singles Chart: http://officialcharts.com. AcousticBrainz: https://acousticbrainz.org.
